# An injectable curcumin-loaded hydrogel for neuroprotective treatment promote nerve tissue repair in rat severe spinal cord injury

**DOI:** 10.3389/fbioe.2025.1655686

**Published:** 2025-09-02

**Authors:** Zhaoqing Zhang, Haiyan Ma, Ruisong Tian, Guangwei Li, Fuchang Zhao, Liangyu Xie, Hao Chen

**Affiliations:** ^1^ Neck-Shoulder and Lumbocrural Pain Hospital of Shandong First Medical University, Shandong First Medical University and Shandong Academy of Medical Sciences, Jinan, China; ^2^ College of Medical Information and Artificial Intelligence, Shandong First Medical University, Jinan, China

**Keywords:** spinal cord injury, curcumin, injectable hydrogel, polyphenol, oxidative stress

## Abstract

**Introduction:**

Spinal Cord Injury (SCI) leads to severe motor and sensory impairments and underscores the urgent need for the development of effective treatment approaches. The injury microenvironment with oxidation, inflammation and extracellular matrix disruption represents a major barrier to nerve tissue repair as well as the functioning of therapeutic factors.

**Methods:**

This study proposes a curcumin-loaded boronate-crosslinked tannic acid injectable hydrogel (CBT-gel) for effective antioxidants and neuroprotective spinal cord repair. Rat transection SCI models were established, and the CBT-gel was implanted in the injured spinal cord tissue. Behavioral and histological testing was performed to determine the spinal cord repair effects.

**Results:**

The injectable self-healing hydrogel induced sustainable release of the neuroprotective drug curcumin. The CBT-gel transplantation treatment enhanced axonal preservation and reduced glial scar formation. The results also revealed a reduction in neuroinflammation and cellular oxidative damage.

**Discussion:**

These findings support the potential of CBT-gel to improve the microenvironment for spinal cord repair by leveraging their antioxidant and neuroprotective properties. The results in this study aligns with the documented bioactivity of curcumin, though molecular targets in the CBT-gel treatment require further profiling.

## 1 Introduction

Spinal Cord Injury (SCI) remains a significant medical challenge, often leading to severe motor, sensory, and autonomic dysfunctions. SCI greatly diminishes the quality of life for individuals affected (Nature, 2023). Current treatment approaches mainly focus on surgical procedures and medication, which, however, usually fail to gain meaningful functional recovery (Yin et al., 2024). After an SCI, a secondary injury process occurs, marked by oxidative stress, inflammation, and cell death, which exacerbates neuronal damage and hinders the spinal cord’s natural ability to heal. Therefore, there is a pressing need for integrated treatment that can effectively reduce the effects of secondary injury and promote nerve repair (Arefnezhad et al., 2024; Hadadi et al., 2025).

Recent advancements in regenerative medicine have highlighted the potential of bioactive compounds in promoting nerve repair, while major challenges often remain in their bioavailability and therapeutic effectiveness. Curcumin is a polyphenolic compound derived from turmeric that has been recognized with diverse neuroprotective properties, including reducing oxidative stress, modulating inflammatory responses, and supporting neuronal survival and regeneration (Pandey et al., 2008; Alvarado-Sanchez et al., 2019). Despite its promise, the potential benefits in treating SCI are limited by the low solubility and rapid metabolism (Prasad et al., 2014). To address this issue, researchers have investigated various delivery systems, such as nanoparticles and hydrogels, aimed at enhancing the stability and controlled release of curcumin at the injury site, ultimately maximizing its therapeutic impact (Ahmad et al., 2024). Injectable hydrogels have become an attractive option for localized drug delivery due to their numerous advantages, including ease of application, biocompatibility, and the ability to adapt to irregularly shaped defects. These hydrogels can be carefully designed to provide a supportive extracellular matrix (ECM) environment, which is essential for cellular regeneration (Li et al., 2022; Lin et al., 2025). By integrating curcumin into a hydrogel matrix, a sustained-release formulation can be developed that not only delivers the therapeutic agent effectively but also simulates the natural extracellular matrix. This enhances cellular interactions and supports neuroregeneration, making it a promising approach in the field of regenerative medicine.

In this study, we investigated the neuroprotective effects of a curcumin-loaded tannic acid (TA)-based borate-crosslinked injectable hydrogel (CBT-gel) in a rat model of complete spinal cord transection. The combination of curcumin’s pharmacological properties with the supportive characteristics of the hydrogel created a synergistic effect, ultimately leading to improved outcomes in spinal cord injury repair. By improving the bioavailability of curcumin and creating a supportive environment for nerve repair, this hydrogel formulation enhances neuronal survival and reduces inflammation. The development of CBT-gel represents a novel therapeutic approach to tackling the complex challenges associated with spinal cord injuries.

## 2 Methods

### 2.1 Reagents and animals

N-(3-dimethylaminopropyl)-N′-ethylcarbodiimide hydrochloride (EDC), Chitosan (CS, Mη 20 k, DDAc 90%), 1-hydroxybenzotriazole (HOBt), 3-carboxyphenylboronic acid (PBA) and TA were purchased from Maclin Co., Ltd. (Shanghai, China). Cellulose dialysis tubing (3.5 k MWCO) was purchased from Yi bo biolocigal Co., Ltd. (Changsha, China). All chemicals were used without further purification. Female Sprague-Dawley (SD) rats weighing between 220–250 g, utilized for the SCI model, were acquired from Beijing Weitong Lihua Experimental Animal Technology Co., Ltd (Beijing, China). Approval of all animal experiments and procedures was granted by the Animal Care and Use Committee (202402029).

### 2.2 Synthesis of CS-PBA

The CS/HOBt and PBA/EDC solutions were prepared separately. To prepare the CS/HOBt solution, chitosan (298 mg, 1.85 mmol) and HOBt (250 mg, 1.85 mmol) were dissolved in 40 mL of deionized water. The mixture was stirred until clear. To prepare the PBA/EDC solution, PBA (307 mg, 1.85 mmol) and EDC (354 mg, 1.85 mmol) were dissolved in 1 mL of DMSO for 6 h using a magnetic stirrer. The PBA/EDC solution was then added dropwise to the CS/HOBt solution, which was stirred with a magnetic stirrer for 24 h. The final mixture was dialyzed in water alternately with dialysis bags (MWCO 3.5 k Da) for 3 days, and at the end of dialysis it was freeze-dried for 48 h. The chemical structure was characterized by using ^1^H nuclear magnetic resonance (^1^H NMR) and Fourier transform infrared spectroscopy (FTIR).

### 2.3 Preparation of curcumin-loaded CS-PBA/TA (CBT) hydrogels in one-pot

Initially, CS-PBA was dissolved in 1% (w/v) acetic acid solution to obtain 6% (w/v) solution and dispersed with curcumin at a concentration of 5 mg/mL. Meanwhile, TA solutions with concentrations of 3% (w/v), 5% (w/v), and 10% (w/v) were prepared according to the volume ratio of 1:1. These solutions were then mixed and blown uniformly into 96-well plates. Thereafter, the plates were left to stand, allowing the solutions to naturally form into gels. These solutions were named CBT1, CBT2, and CBT3, respectively. The chemical structures of the solutions were characterized via FTIR.

The compatibility of hydrogel materials was assessed using the PC12 cell line. Initially, PC12 cells were cultured in DMEM/F12 medium supplemented with 10% fetal bovine serum (FBS) and were plated in 96-well plates for 24 h before the experiment. To ensure sterility, the hydrogel was exposed to ultraviolet (UV) light and immersed in ethanol. Hydrogel extracts were then prepared by soaking the hydrogel in cell culture medium for 72 h. After discarding the original culture medium, the cells in 96-well plates were either cultured in the standard complete medium or cultured in complete medium enriched with the hydrogel extract at different concentrations. Following a 24 h incubation period, the viability of the PC12 cells was assessed using the Cell Counting Kit-8 (CCK-8) assay.

### 2.4 *In vitro* drug release performance testing

The CBT-gel was soaked and incubated in 500 μL PBS at 37 °C. After a certain time interval, 50 μL of PBS solution was collected and replenished with an equal volume of PBS solution. The concentration of curcumin was measured at 426 nm and the drug release rate curve was plotted.

### 2.5 Surgery for spinal cord transection and CBT-gel implantation

The rat SCI model was established through creating a transection lesion. Rats were randomized into three groups including Sham (n = 6), SCI + PBS (n = 8), SCI + CBT-gel (n = 8). Under deep anesthesia, hair was removed from the back of the rats near the T10 spinous process. Following muscle separation, a laminectomy was performed to access the dorsal surface of the T9-11 segment. A complete transection of the T9-T10 spinal cord segment resulted in a lesion gap measuring 2.0 mm. Following meticulous hemostasis, the CBT-gel of a volume of 20 μL was implanted through an injection to precisely fit the spinal cord gap. Phosphate-buffered saline (PBS) solution was also injected to establish a non-treated SCI group. The musculature and skin were subsequently sutured. To prevent infection, penicillin was administered for 7 days post-operation. For postoperative care, manual bladder massage was performed twice daily until reflexive bladder control was restored. Rat locomotor functionality following SCI modeling was assessed using the 21-point Basso-Beattie-Bresnahan (BBB) locomotion rating scale. The subjects permitted unrestricted movement, while observers, unaware of the assigned groups, conducted the assessments. The analysis focused on the stepping movements and the coordination exhibited between the forelimbs and hindlimbs.

### 2.6 Tissue processing and immunohistochemistry

Animals were perfused under deep anesthesia using isotonic physiological saline, followed by a fixation with 4% paraformaldehyde in PBS. Histological quantification was performed by two investigators blinded to group assignments using randomized coded samples. For the purpose of immunofluorescence staining of the spinal cord, the harvested spinal cord tissues were embedded and subsequently cryosectioned into slices of 16 μm thickness. Following fixation in acetone at 4 °C, the sections were incubated overnight at 4 °C with specific primary antibodies targeting neurofilament (NF) (Proteintech, China), glial fibrillary acidic protein (GFAP) (Boster, China), et al. After washing, the sections were treated with secondary antibodies at 37 °C for 1 hour. Nuclei were stained with DAPI (Proteintech, China). Observations were conducted using laser scanning confocal microscopy (N1R, Nikon, Japan), and quantitative analysis was carried out using ImageJ.

### 2.7 Statistical analysis

The quantitative results are presented as mean ± standard deviation (SD). Statistical analyses were performed using GraphPad Prism version 9.0. In instances where normal distribution and homogeneity of variance were confirmed, a two-tailed unpaired t-test was employed. For data exhibiting non-normal distribution, the Mann-Whitney U test was utilized. A P value of less than 0.05 was considered indicative of statistical significance. Additional analyses were conducted.

## 3 Results

### 3.1 Characterization of the hydrogel

**FIGURE 1:**
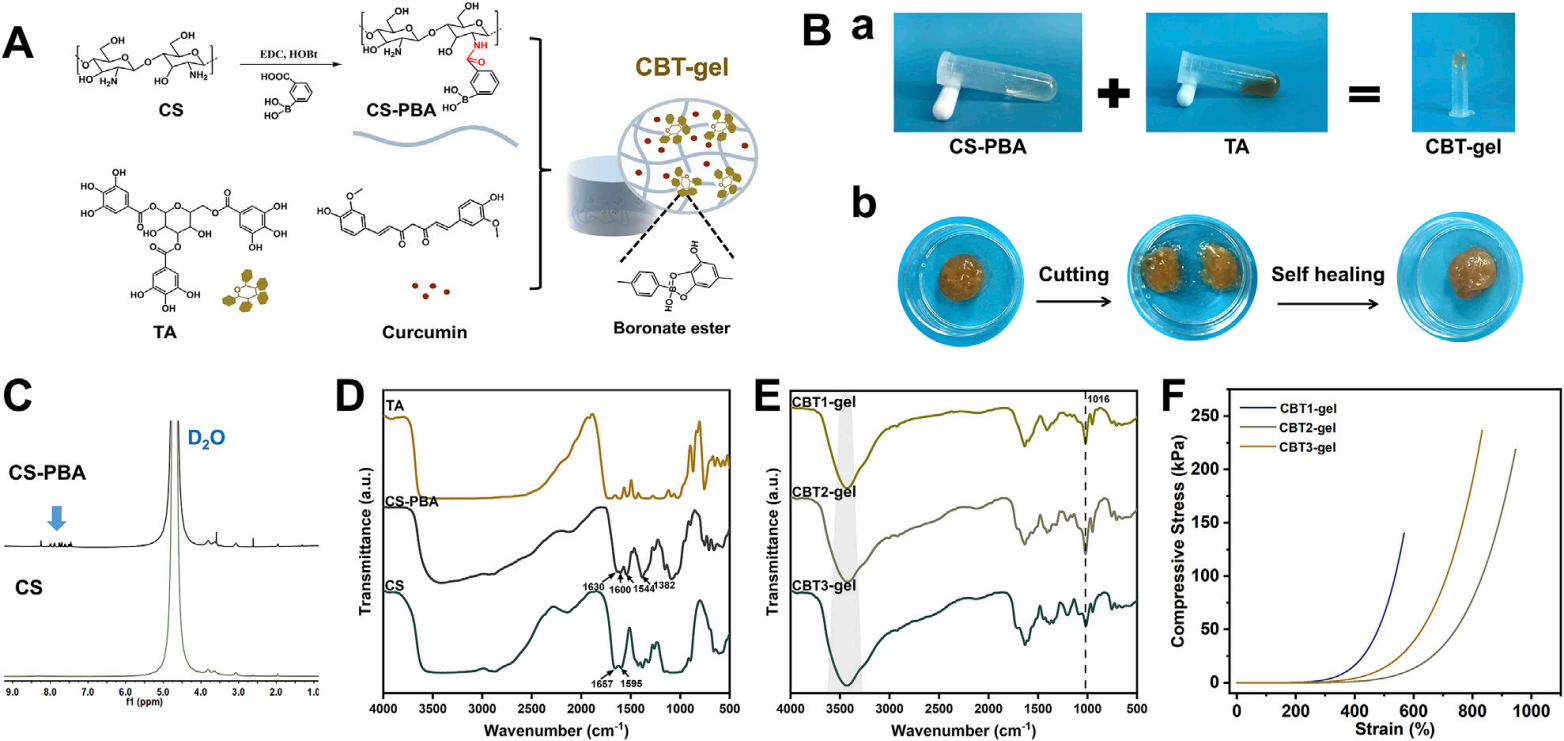
**(A)** Synthesis of CS-PBA, CBT hydrogel and the mechanism of hydrogel formation. **(B)** Hydrogel properties before and after cross-linking and self-healing behavior of hydrogels. **(C)** 1H NMR spectrum of CS, and CS-PBA. **(D)** FTIR spectra of CS, CS-PBA, and TA. **(E)** FTIR spectra of CBT1, CBT2 and CBT3 hydrogels. **(F)** Representative compressive stress-strain curves for CBT hydrogels with different concentrations.

Figure 1A demonstrates the synthesis of CS-PBA, CBT hydrogel and the mechanism of hydrogel formation. In the CBT hydrogel, CS-PBA and TA were crosslinked by dynamic borate bonding and intermolecular weak hydrogen bonding to form a chemical and physical dual network structure. Figure 1B shows the gelation effect of CBT hydrogels and their self-healing properties. After gelation, the CBT hydrogel does not flow even when inverted, indicating excellent gelation performance. In addition, when the two CBT hydrogel samples were cut off by external force, they were able to re-heal after 60s of resting, indicating that the presence of borate bonding gives CBT hydrogels good self-healing behavior. The hydrogel showed resonance characteristic peaks of aromatic ring structure in the range of 7.86 to 7.79 ppm in the ^1^H NMR of CS-PBA (Figure 1C), indicating the successful conjugation of PBA with chitosan. In Figure 1D, the FTIR image shows that the absorption peaks at the wave numbers 1650–1590 cm^-1^ indicate the presence of bending vibrations of the primary amine moiety N-H. After grafting, the absorption peaks here were shifted or weakened due to the amidation reaction of -NH_2_ of CS with -COOH of PBA. The FTIR spectra of CS-PBA showed characteristic absorption peaks at 1382 cm^-1^ for -B(OH)_2_ group and at wavelength 1544 cm^-1^ for benzene ring, which further proved that the grafting reaction occurred successfully. Following the cross-linking of CS-PBA with TA in CBT hydrogels to form gels, FTIR spectra of CBT hydrogels (Figure 1E) demonstrated that the intensity of the stretching vibration absorption peak of -OH was significantly enhanced, while the characteristic absorption peak of the -B(OH)_2_ groups were weakened accordingly. Concurrently, new absorption peak was detected at 1016 cm^-1^. This outcome can be ascribed to the establishment of a borate bond. The result thus confirmed the effective cross-linking between CS, PBA and TA. Subsequent studies demonstrated that the intensity of the -OH stretching vibrational absorption peaks exhibited a gradual enhancement with increasing TA concentrations (Figure 1E). This suggests that an increase in TA concentration promotes an increase in the degree of cross - linking between CS-PBA and TA, which leads to a corresponding change in the vibrational properties of -OH. This is further verification that the degree of cross-linking reaction is affected by the TA concentration. Consequently, it can be deduced that the enhanced cross-linking network may exert significant effects on the overall properties of hydrogels, including an augmentation in their mechanical strength and a modification in their swelling behavior. The compressive strengths of the hydrogels were examined using a universal testing machine (Figure 1F). It was demonstrated that the compressive strength of the CBT hydrogels increased from 140 kPa to 236 kPa with increasing TA concentrations. The compressive strengths of the CBT2 hydrogels were almost comparable to those of CBT3. Furthermore, the CBT2 hydrogel exhibited optimal ductility. CBT2 was selected for the *in vivo* implantation due to optimal ductility and compressive strength, minimizing mechanical mismatch with spinal tissue. These findings suggest that the mechanical properties of hydrogels can be modulated by adjusting the TA concentration.

**FIGURE 2:**
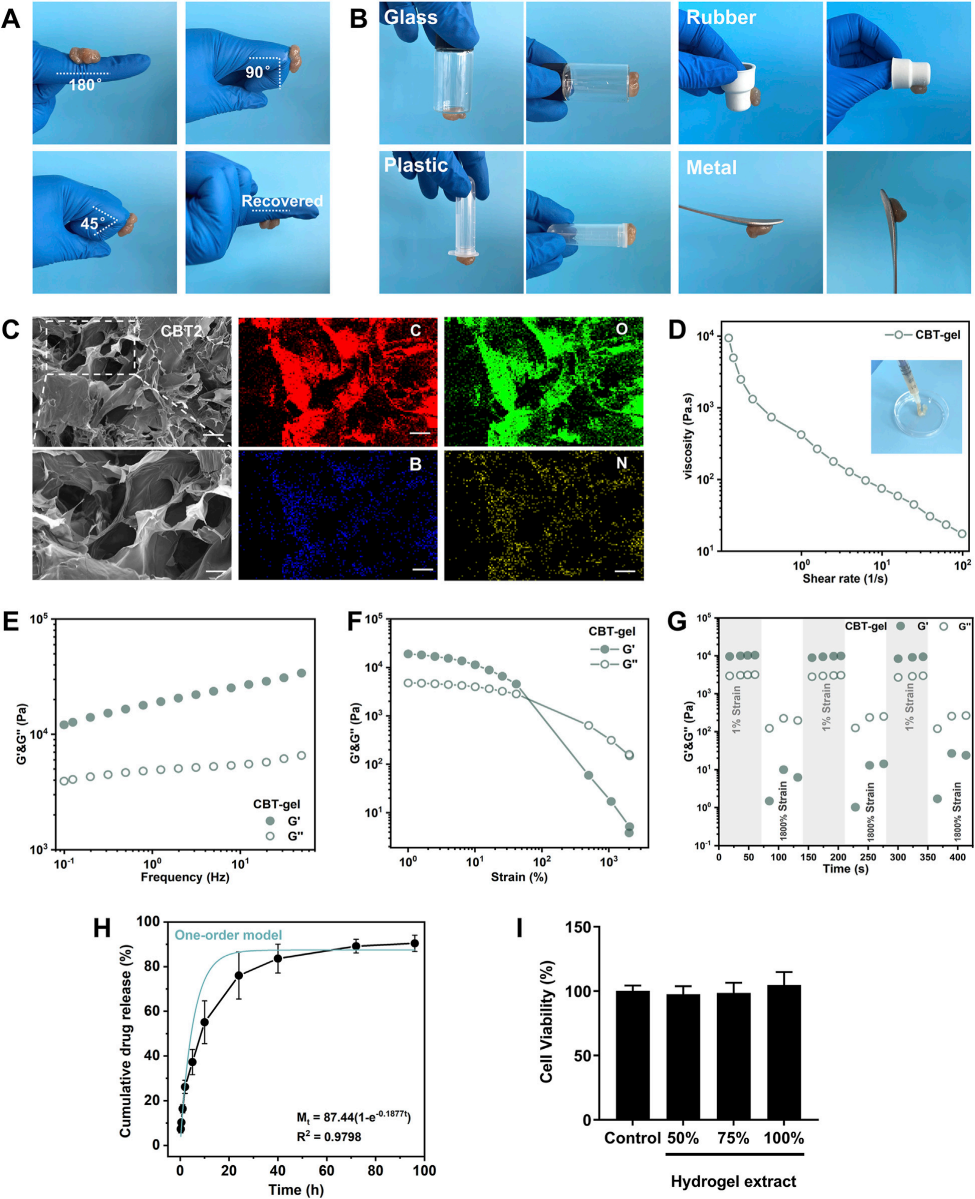
**(A)** Hydrogel adhering to finger, bending at an intended angle the hydrogel does not fall off and can be restored to its original state. **(B)** Hydrogel adhesion to glass, rubber, plastic, and glass surfaces. **(C)** SEM images of CBT hydrogels and EDS images of hydrogels. Scale bars: 100 μm (unzoomed in C), 20 μm (zoomed in C). **(D)** Shear thinning experiment conducted at 25 °C with a variable shear rate of 10^–1^–10^2^ s^−1^. **(E)** Frequency sweep of the hydrogel at 25 °C under 1% constant strain, covering a range of angular frequencies from 0.1 to 50 rad s^−1^. **(F)** Stress sweep of the hydrogel at 25 °C under 10 rad s^−1^ constant angular frequency, with strain ranging from 0.1% to 1000%. **(G)** Alternating step stress test of the hydrogel at 25 °C, with alternating strains of 1% and 1800% over 60s at a constant angular frequency of 10 rad s^−1^, yielding the storage modulus (G′) and loss modulus (G″). **(H)** The release profile of curcumin from the hydrogel. The blue curve indicates the kinetic model of one-order model of curcumin release. **(I)** Viability of PC12 cells incubated with the hydrogel extracts in different concentrations for 24 h. Data are presented as mean ± SD.

The adhesion, stretching and compressive properties of hydrogels are critical to the effectiveness of tissue repair. The synergistic cross-linking mechanism combines the advantages of physical and chemical cross-linking to improve adhesion and compressive properties of the hydrogel. Physical cross-linking provides dynamic adaptation and stress dispersion to enhance mechanical strength, while chemical cross-linking strengthens the structure, enhances adhesion and ensures long-term stability. The resultant hydrogels exhibit excellent adhesion in wet environments. The polyphenolic groups of TA incorporated in the hydrogel interacted strongly with the sulfhydryl and amino groups on the tissue surface, significantly enhancing its adhesion. Hydrogels are able to adapt to the lesion and can recover deformations (Figure 2A). Figure 2B shows that the CBT hydrogel has good adhesion properties and can spontaneously adhere to the surface of materials such as glass, plastic, rubber, and metal. Figure 2C, SEM images demonstrate that CBT hydrogel presents rich and porous three-dimensional network structures, which are useful for absorbing tissue hemorrhage to promote the tissue repair process. Meanwhile, from the elemental analysis images, the hydrogel was rich in boron, which could prove the successful grafting of phenylboronic acid. In addition, the porous structure provides a large surface area for cell attachment and growth, which is conducive to cell proliferation and migration, accelerating the regeneration and repair of injured tissues.

As seen on Figure 2D, the viscosity of the CBT hydrogel was subsequently changed when the shear rate was in the range of 0.1–100 s^-1^. The results show that the hydrogel exhibits significant non-Newtonian fluid properties, with obvious shear-thinning behavior, and at the same time possesses a certain degree of fluidity: the CBT hydrogel can be smoothly extruded through a syringe. The viscoelasticity and structural recovery behavior of the CBT hydrogels were investigated by rheological tests. In order to characterize the viscoelastic properties of the hydrogels, frequency scanning experiments (Figure 2E) were carried out at a constant strain of 1% at a temperature of 25 °C, with a range of angular frequencies from 0.1 to 50 rad s^-1^. The results show that the energy storage modulus (G′) is always greater than the loss modulus (G″) over the frequency range scanned, which indicates that the hydrogel maintains its gel-like structure even at high frequencies. Subsequently, strain amplitude scans were carried out at a constant angular frequency of 10 rad s^-1^, with a strain range from 1% to 2000%, to investigate the relationship between the changes in G′ and G”. Figure 2F shows that at low strains, both G′ and G” remain stable and G′ is always larger than G″, which indicates that the hydrogel is in a stable gel state. With the gradual increase of strain, when the strain reaches 500%, the intersection of G′ and G″ occurs, which marks the transition of hydrogel from the gel state to the sol state. The results show that the hydrogel exhibits excellent strain tolerance, which makes it capable of withstanding mechanical strains that may occur during the tissue repair process.

Finally, alternating step strain tests were performed to assess the rheological recovery behavior of hydrogels under alternating strains. The results of Figure 2G show a significant decrease in G′ values at high strains (1800%) when alternating strains of 1% and 1800% were applied, with each strain stage lasting 60 s. This indicates that the hydrogel has a good recovery potential despite its structural damage after being subjected to extreme strains, proving self-healing properties. The self-healing properties could prevent fragmentation under spinal movement, ensuring continuous therapeutic coverage in dynamic injury cavities. The release of curcumin from this hydrogel system lasted for more than 96 h, with a final cumulative release ratio of over 80% (Figure 2H). Curcumin release best fit one-order model (*R* ^2^ = 0.89), indicating diffusion-dominated release. This sustained release profile would benefit to modulate the acute oxidative stress and neuroinflammation after implantation in the injured spinal cord. Finally, the hydrogel materials were assessed in PC12 cells and were biocompatible with the neural cells (Figure 2I).

### 3.2 The CBT-gel treatment facilitated spinal cord nerve repair

**FIGURE 3:**
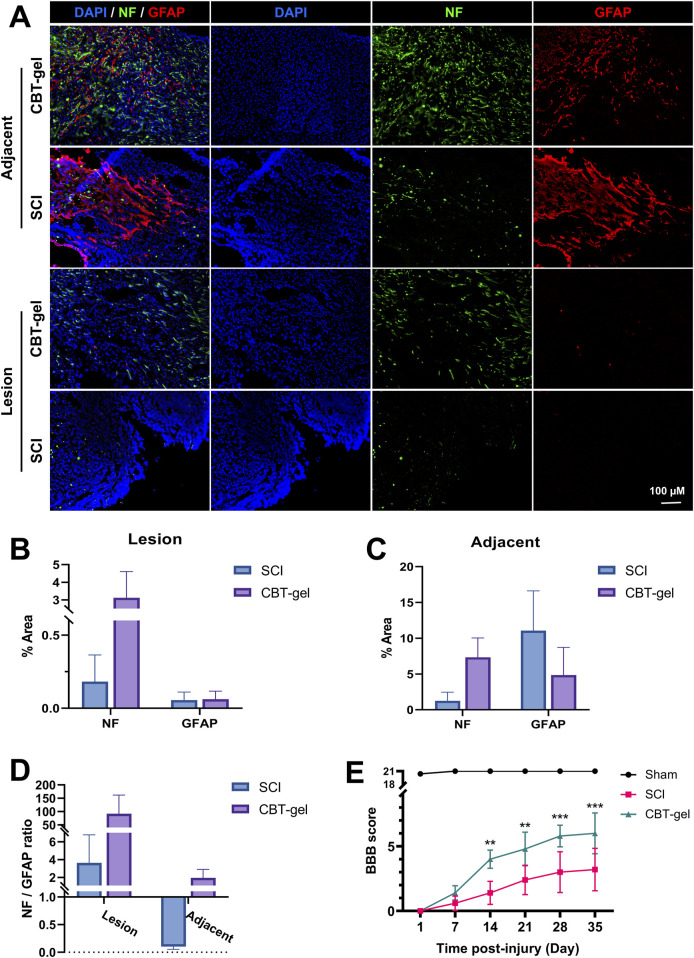
Evaluation of NF and GFAP in the spinal cord tissues and assessment of behavior function. **(A)** Representative fluorescent micrographs of immunofluorescence staining of NF (green) and GFAP (red) at Day 35 in spinal cord tissues of CBT-gel treatment and SCI group. Nuclei were stained by DAPI (Blue). **(B–D)** Tissues from the lesion epicenter **(B)** and adjacent **(C)** regions were quantified for positive areas of NF and GFAP, and the NF/GFAP ratios were calculated **(D)**. NF/GFAP ratio = (NF + area)/(GFAP + area in same region). **(E)** BBB scores of the animals during the 35-day recovery. Data are presented as mean and SD (n = 6–8). Statistical analysis was assessed by Mann-Whitney U test *p < 0.05, **p < 0.01, ***p < 0.001.

The effects of CBT-gel implantation on nerve tissue regeneration were assessed by evaluating the distribution of neurofilament (NF) and glial fibrillary acidic protein (GFAP). The tissue treated with CBT-gel showed a reduction in lesion cavity size and an increase in NF^+^ nerve fibers (Figure 3A). The GFAP^+^ astrocytes, which serve as indicators of glial scars, showed more loosely structured patterns in the treatment group compared with SCI group. Following treatment, the density ratios of NF^+^ to GFAP^+^ cells in the CBT-gel group were significantly altered (Figures 3B–D). In the SCI group, GFAP^+^ cell aggregation was noted at the lesion margin, resulting in a poorly repaired cavity. In contrast, the spinal cord repaired with CBT-gel presented significantly elevated densities of nerve fibers across various segments. The significant increase in NF and GFAP expression suggests that the curcumin-loaded hydrogel plays a dual role in promoting neuronal repair while simultaneously preventing the formation of glial scars, which are recognized as barriers to regeneration after spinal cord injury. After 2 weeks of treatment, the CBT-gel group exhibited significant improvement in motor functions compared with the SCI group (Figure 3E), and this therapeutic effect was maintained became more pronounced in the subsequent recovery process. Finally, after 5 weeks of recovery, the BBB scores in the CBT-gel groups reached around six points. As the histological improvements indicated the reparative potential of the CBT-gel, the functional assessments of BBB scores could further suggest clinical correlation.

**FIGURE 4:**
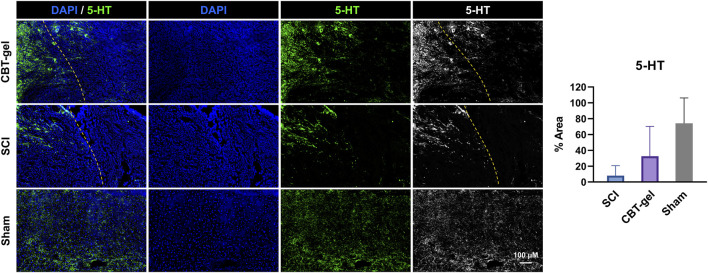
Investigation of 5-HT distribution in the injured spinal cord tissues in CBT-gel, SCI and sham groups. The dashed lines indicated the rostral margins of the lesion. Nuclei were stained by DAPI (Blue), and 5-HT was stained in green. Positive areas of 5-HT staining were quantified.

The CBT-gel group exhibited a significant increase in 5-HT immunoreactivity compared to the untreated spinal cord tissues. Histological analysis revealed that 5-HT-positive nerve fibers extended across the lesion boundary and infiltrated the injury core in CBT-gel-treated animals, suggesting enhanced serotonergic axon regeneration (Figure 4). In contrast, untreated SCI tissues showed sparse 5-HT staining restricted to regions distal to the injury site. As 5-HT is a critical neurotransmitter for locomotor circuitry and descending motor pathway plasticity, these findings imply that the hydrogel-mediated delivery of curcumin facilitates neural repair by promoting the regrowth of neuromodulatory axons into the damaged microenvironment.

### 3.3 The CBT-gel treatment alleviated oxidative and inflammatory microenvironment

**FIGURE 5:**
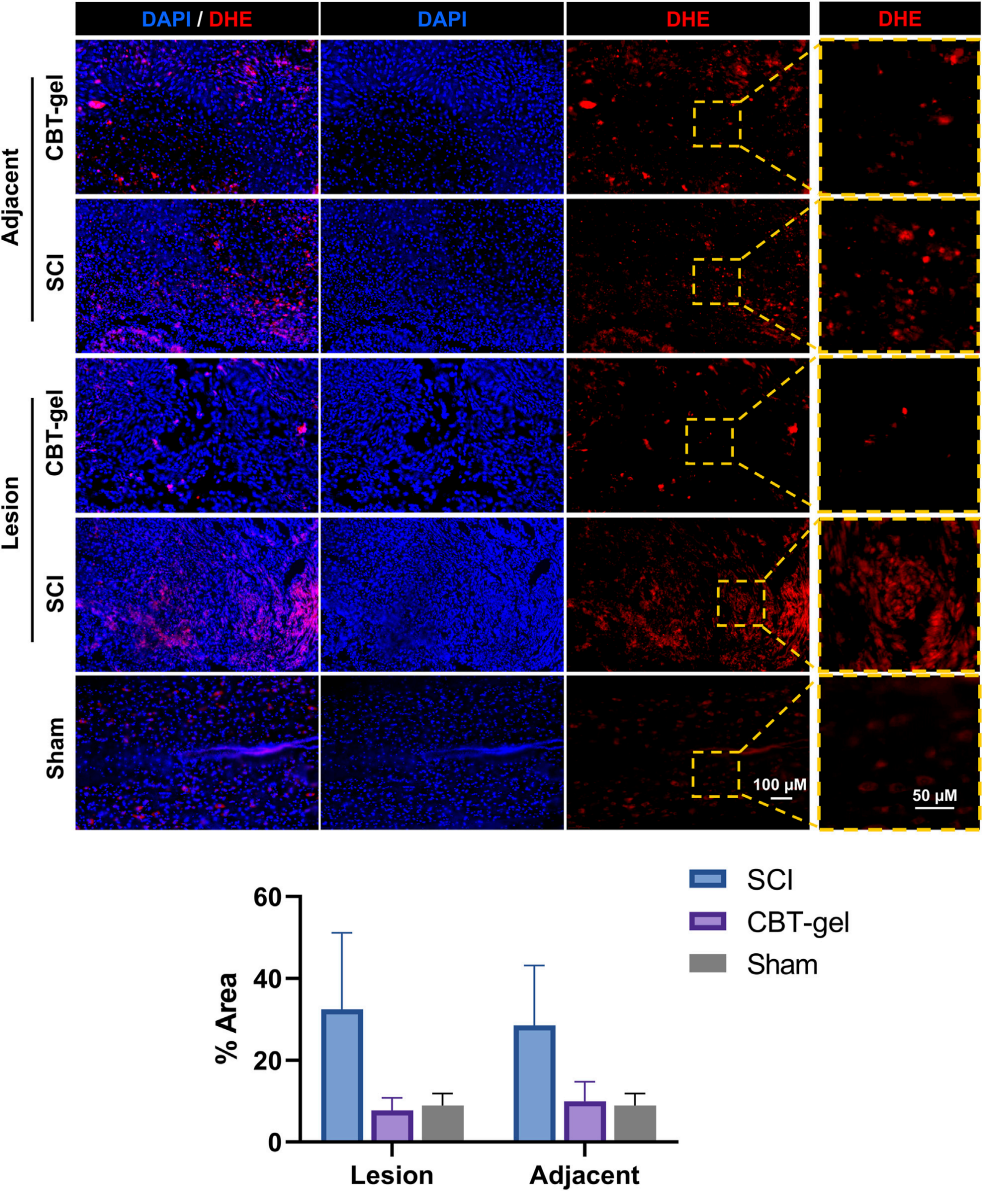
Representative fluorescent micrographs and quantification of immunofluorescence staining of DHE (red) at Day 35 in spinal cord tissues of CBT-gel, SCI and sham groups. Nuclei were stained by DAPI (Blue). In CBT-gel and SCI groups, tissues from the lesion epicenter and adjacent regions were evaluated.

DHE staining, a marker of reactive oxygen species (ROS), demonstrated a marked reduction in oxidative stress within the treated group (Figure 5). This attenuation of ROS accumulation aligns with the known antioxidant properties of curcumin, which likely scavenged free radicals and mitigated secondary oxidative damage post-SCI. The diminished DHE signal could correlate with improved tissue preservation in the treatment group. This result underscored the therapeutic potential of curcumin-hydrogel composites in ameliorating oxidative stress after the neural trauma.

**FIGURE 6:**
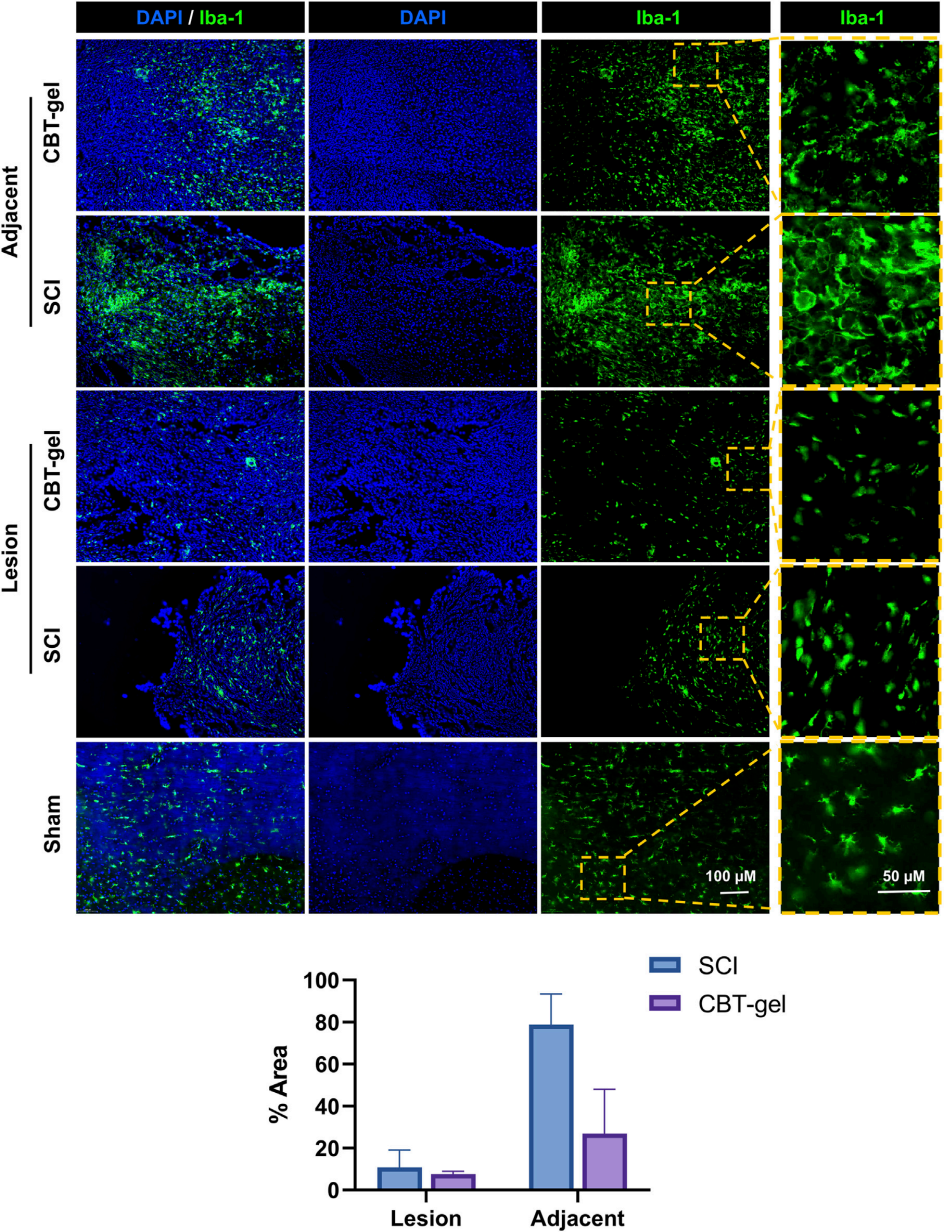
Investigation on the distribution and morphologies of Iba-1 (stained green) positive cells in the injured spinal cord tissues in CBT-gel, SCI and sham groups. The boxed regions were zoomed and showed on the right panels for the detailed morphologies of the cells. Nuclei were stained by DAPI (Blue). Staining areas of Iba-1 in SCI and CBT-gel groups were quantified in lesion and adjacent regions.

Iba-1 immunostaining, indicative of activated microglia and infiltrating macrophages, showed a decrease in cell density within the lesion epicenter as well as the adjacent tissues of the treatment group compared to untreated SCI. The CBT-gel intervention not only reduced Iba-1^+^ cell numbers but also suppressed their morphological activation with fewer amoeboid-shaped cells (Figure 6), suggesting a transition from pro-inflammatory to reparative phenotypes. These results highlight the hydrogel’s anti-inflammatory efficacy. Given that sustained neuroinflammation exacerbates tissue damage post-SCI, this modulation of immune responses may create a permissive microenvironment for neural repair.

## 4 Discussion

SCI poses a significant challenge in clinical and research environments due to its intricate pathophysiology and the severe effects on patients’ quality of life. After the initial traumatic event, various secondary injury mechanisms worsen the damage and impede recovery, such as oxidative stress, inflammation, and neuronal apoptosis. These detrimental processes contribute to considerable motor, sensory, and autonomic dysfunction, ultimately leading to long-term disability. There is an urgent need for effective therapeutic strategies in promoting neurological recovery and functional rehabilitation.

In this study, we explored the neuroprotective effects of an injectable curcumin-loaded hydrogel using a rat model of complete spinal cord transection. Although contusions are considered better replicate human SCI pathophysiology, complete transection eliminates the confounding effects of spared tissue, enabling clear assessment of axonal regeneration across defined gaps. And this method serves as an established approach for hydrogel screening (Li L. et al., 2019). Another significant reason for utilizing the complete transection model is the remarkable self-healing capabilities observed in rodents. Compared to humans, the enhanced self-healing ability of spinal cord tissue in rodents after contusion injury can create a substantial disparity between the challenges faced in repair treatments and the actual clinical scenarios encountered in human patients. Our results indicate that the curcumin hydrogel played a crucial role in neuroprotection and inflammation regulation.

Curcumin has been previously reported to play a crucial role in influencing key signaling pathways, notably the nuclear factor kappa B (NF-κB) pathway (Gu et al., 2023), which serves as a key regulator in reducing neuroinflammation (He et al., 2025; Wu et al., 2024). These findings provide a strong foundation for investigating curcumin as a potential treatment for SCI. In the current study, the reduction in Iba-1 expression observed in the treatment group suggests a decrease in microglial activation. Suppression of microglial NF-κB activation by curcumin could reduce TNF-α/IL-1β (Li X. et al., 2019). This also explains the attenuated Iba1+ activation and morphological shifts in this study. The immunomodulatory effect of curcumin aligns well with previously reported properties, indicating its potential to create an environment that supports neuronal survival and regeneration (Namgyal et al., 2020; Drion et al., 2018; Dairam et al., 2007). Additionally, the observed decrease in oxidative stress, as evidenced by DHE staining, further highlights curcumin’s antioxidant capabilities. This is crucial for protecting neurons from apoptosis and enhancing overall cellular health (Li L. et al., 2019). The reduced Iba1+ activation and DHE intensity provided direct evidence of attenuated neuroinflammation/oxidative stress, which aligns with the documented bioactivity of curcumin (Li X. et al., 2019; Bharathiraja et al., 2023), though molecular targets require further profiling. Collectively, these insights highlight the importance of curcumin in promoting beneficial effective recovery after SCI.

In recent years, curcumin-loaded hydrogels were reported to promote SCI repair. For instance, Chen et al. (2024) showed that a decellularized spinal cord matrix hydrogel, when combined with neurotrophin-3 (NT-3) and curcumin, significantly enhanced the proliferation and differentiation of neural stem cells. This combination not only reduced inflammation but also led to improved functional recovery in rat models of SCI, highlighting the synergistic effects of these agents in modifying the injury microenvironment (Chen et al., 2024). Similarly, Ai et al. (2023) examined a gelatin/alginate hydrogel scaffold infused with curcumin and human endometrial stem cells, finding that this combination facilitated spinal cord regeneration in rats, as evidenced by better histological outcomes and functional recovery (Ai et al., 2023). In another study, Tan et al. (2024) created a bioinspired hydrogel that incorporated hyaluronic acid and a designer peptide, which, when combined with NT-3 and curcumin, promoted axonal regrowth and functional recovery in both rodent and canine models of SCI. This research underscored the hydrogel’s capability to guide neuronal connections and enhance locomotor function following injury (Tan et al., 2024). Lajmiri et al. (2024) investigated a chitosan hydrogel loaded with selenium nanoparticles and curcumin, revealing that this delivery system significantly decreased inflammation and fostered neuroprotection in a rat model of SCI, demonstrating the promise of combined therapies for improving recovery (Lajmiri et al., 2024). A curcumin-loaded dynamic hydrogel aimed at treating chronic peripheral neuropathy was demonstrated to reduce pain and enhance locomotor function, indicating a potential application for curcumin in addressing nerve injuries (Kong et al., 2023). Similarly, Elkhenany et al. (2021) developed a hyaluronic acid-based scaffold that incorporated curcumin along with human neural precursor cells. This approach provided neuroprotection and minimized fibrotic invasion in SCI models, showcasing a comprehensive strategy for promoting tissue regeneration (Elkhenany et al., 2021). In another study, Luo et al. (2021) introduced a self-healing hydrogel capable of releasing curcumin in a controlled manner, which significantly improved Schwann cell migration and neurite outgrowth *in vitro*, ultimately leading to better functional recovery in SCI models (Luo et al., 2022). Furthermore, Liu et al. (2024) performed a systematic review of biomaterials infused with traditional Chinese medicine components, including curcumin. This review highlighted the effectiveness of these materials in fostering neuroprotection and functional recovery in SCI, while also calling for additional research to refine drug delivery systems (Liu et al., 2024). Collectively, previous studies in recent years have highlighted the promising potential of curcumin-loaded hydrogels in SCI repair, demonstrating their capacity to modify the injury microenvironment, enhance neuroprotection, and support functional recovery.

In this study, unlike single-network hydrogels, the dual-crosslinked CBT-gel uniquely combines several advantages: (a) pH-responsive drug release via boronate ester hydrolysis (Zhou et al., 2025); (b) TA-mediated ROS scavenging independent of curcumin (Cao et al., 2023); (c) self-healing for mechanical stability in dynamic spinal tissue (Hogan et al., 2018). Adhesion properties were qualitatively validated and aligned with TA-polyphenol tissue adhesion mechanisms (Zhang et al., 2021). All components of the hydrogel including chitosan, TA, PBA are FDA-approved for medical devices. No signs of infection/tissue necrosis were observed histologically (Figures 3–6). Prior studies confirm under physiological conditions biodegradation of boronate hydrogels within several hours or days (Terriac et al., 2024). On the other hand, the study lacks an empty hydrogel control group. While prior studies indicate chitosan-TA hydrogels exert mild anti-inflammatory effects (Zhang et al., 2021), future work will decouple scaffold *versus* drug contributions.

The implications of this research extend to the creation of new therapeutic strategies that utilize the unique characteristics of curcumin-loaded hydrogels. By successfully integrating curcumin into a biodegradable hydrogel matrix, we can achieve sustained drug release while also providing an environment similar to the neural ECM. This supportive setting not only improves cell survival but also promotes axonal growth, highlighting the potential of these hydrogels in advancing treatment options. The mechanical properties and biocompatibility of hydrogels make them a promising option for clinical applications in treating SCI. The combination of advancements in biomaterial design with the pharmacological advantages of curcumin suggests a viable pathway for tackling the challenges associated with SCI repair. Future research could focus on optimizing hydrogel formulations and assessing their effectiveness in clinical settings, which could potentially transform treatment options for SCI patients.

## 5 Conclusion

In conclusion, our research highlights the effectiveness of curcumin-loaded injectable hydrogels in repairing spinal cords in rats. The CBT-gel effectively regulated the local microenvironment of SCI through the release of curcumin and the replenishment of ECM. The therapeutic application of this strategy significantly enhanced spinal cord regeneration. This research proposes an innovative framework for the localized delivery of curcumin in SCI treatment, which may also inspire future strategies for managing the pathological microenvironment associated with CNS conditions and traumatic injuries. In conclusion, this strategy shows great promise for advancing clinical methods aimed at enhancing functional recovery in patients with spinal cord injuries. Future research will be crucial to validate these findings and further explore the translational potential of this innovative treatment modality.

## Data Availability

The raw data supporting the conclusions of this article will be made available by the authors, without undue reservation.
